# On the Formation and Propagation of Cancerous Cells in the Vicinity of Tumours of the Same Nature

**Published:** 1856-01

**Authors:** 


*On the Formation and Propagation of Cancerouz Cells in the Vicinity of Tumours of the same Nature.* By M. SCHRODER VAN DER KOLK.

The author has studied with much care the mode of propagation of cancerous tumours, whether epithelial or otherwise, with the assistance, of course, of the microscope. He has obtained some results of practical importance, and which should not be lost sight of when the extirpation of cancerous tumours is in question. As the substance of his essay is pretty nearly recapitulated in a few propositions, we shall reproduce them *verbatim*:—

1. In consequence of nutritive interchanges between the cancer cells and the intercellular fluid, the latter acquires the property of producing new nuclei and new cells.

2. This intercellular fluid enters into communication with that which moistens the sound parts situated in the neighborhood of the tumour, and imparts to it the property of producing in its turn similar cells, which thenceforth appear in the tissues hitherto sound, diffusing themselves along the connective tissue.

3. The minuteness and small number of these new cells prevent the discovery of their presence by the naked eye, so much so that the organs contiguous to the tumour may appear to be perfectly sound, although they bear in them the germ of a new cancerous formation.

4. It is therefore important, not only to excise a tolerably large portion of the sound parts, but also to examine with the microscope the tissue which forms the edge of the wound, to see whether this tissue does not contain cancer cells in process of formation.

5. The existence of burning and lancinating pains in the carcinoma may be regarded as an indication of the propagation of the cancerous cells around the tumour to the neighboring nerves; in this case the disease can scarcely be considered as local, or the operation be attempted with any chance of success.

6. The absorption, by the lymphatics and by the veins, of the infected fluid, poisons, to a greater or less degree, the entire body, so that secondary cancers are produced at points far removed from the primary focus; in this case the question of operation could scarcely be any longer entertained.

7. This infected parenchymatous liquid penetrates into the tissues which it moistens, between the sarcolemma of the muscular fibres, the neurilemma of the nerves, &c. These latter investing tubes also absorb the fluid, so that cells may be produced in the interior even of the sarcolemma and of the neurilemma, with the destruction of the muscular or nervous elements included in these membranes.—*Ibid, from Gazette Médicale de Paris*, October 27, 1855.

